# Supplementary material for: High-Resolution Laminar Identification in Macaque Primary Visual Cortex Using Neuropixels Probes
Source: bioRxiv. 2024 Jan 25:2024.01.23.576944. Preprint. [Version 2] doi: 10.1101/2024.01.23.576944 (PMC10849622; doi:10.1101/2024.01.23.576944)
Supplement: Supplementary file 1 [file NIHPP2024.01.23.576944v2-supplement-1.pdf]

bioRxiv preprint doi: https://doi.org/10.1101/2024.01.23.576944; this version posted January 25, 2024. The copyright holder for this preprint (which was not certified by peer review) is the author/funder, who has granted bioRxiv a license to display the preprint in perpetuity. It is made available under aCC-BY 4.0 International license.

High-Resolution Laminar Identification in Macaque Primary Visual Cortex Using Neuropixels Probes

Li A. Zhang, et al.

Corresponding author Email: callaway@salk.edu

This PDF file includes:

Figs. S1 to S10

bioRxiv preprint doi: https://doi.org/10.1101/2024.01.23.576944; this version posted January 25, 2024. The copyright holder for this preprint (which was not certified by peer review) is the author/funder, who has granted bioRxiv a license to display the preprint in perpetuity. It is made available under aCC-BY 4.0 International license.

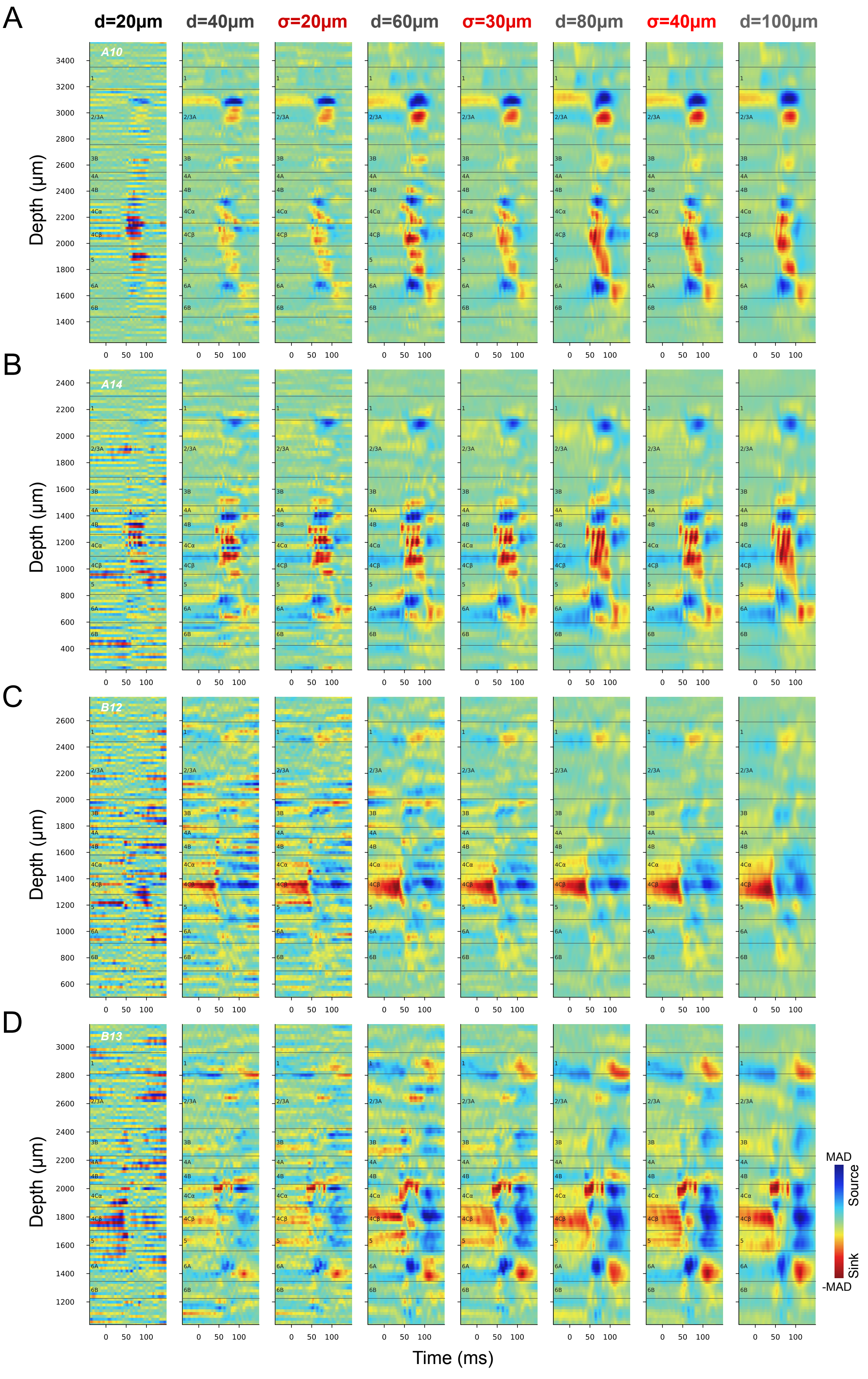

bioRxiv preprint doi: https://doi.org/10.1101/2024.01.23.576944; this version posted January 25, 2024. The copyright holder for this preprint (which was not certified by peer review) is the author/funder, who has granted bioRxiv a license to display the preprint in perpetuity. It is made available under aCC-BY 4.0 International license.

Figure S1. **Comparing spatial averaging to downsampling for CSD profiles evoked by flipping the black screen to the white screen through the dominant eye**. The same four penetrations in Figure 2E-H are shown here (A: A10, B: A14, C: B12, D: B13). In the downsampling process, LFPs from electrodes with increasing vertical spacing (d=20, 40, 60, 80, 100µm, gray) were used to calculate CSDs, then they were interpolated (Cubic Spline) to the vertical resolution of 20µm. In the spatial averaging process, CSD profiles of 20µm spacing were Gaussian filtered with increasing width (σ=20, 30, 40µm, red). The CSD profiles were independently color-mapped according to the maximum absolute deviation (MAD) in each profile.

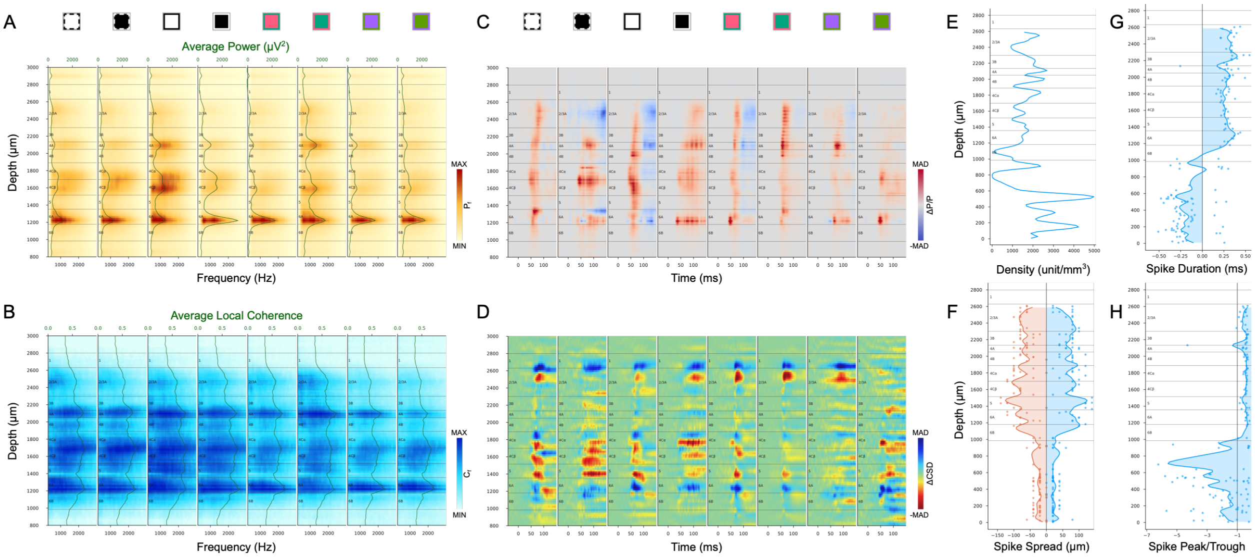

Figure S2. **V1 layer delineation for example penetration (A3) based on power spectrum (A), local coherence (B), instantiations power changes (C), CSD changes (D), unit density (E), spike spread (F), spike duration (G) and spike peak-trough ratio (H).**

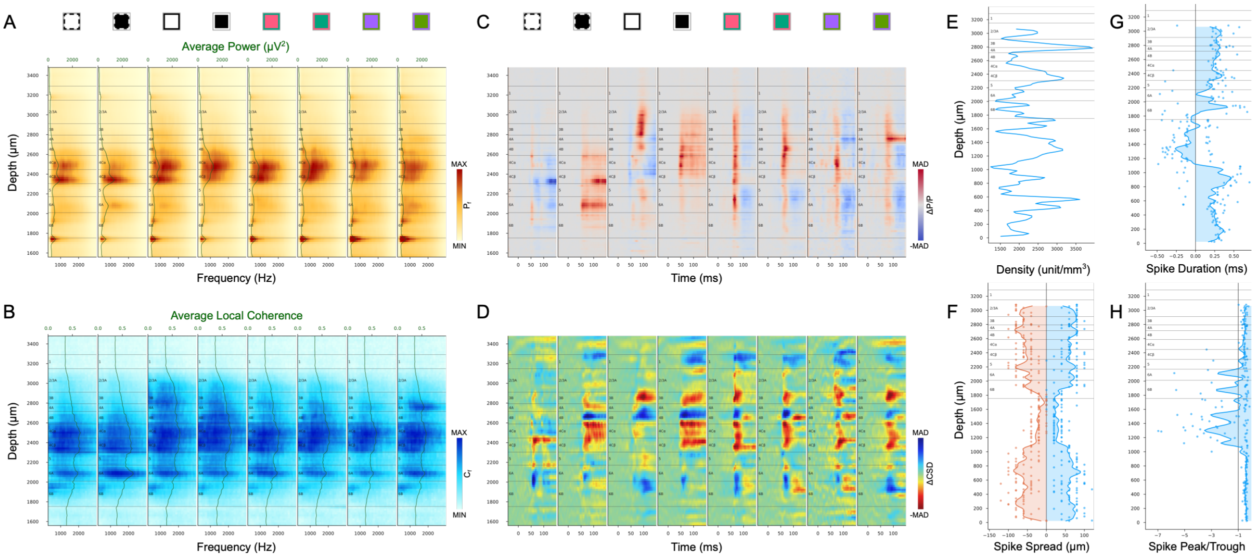

bioRxiv preprint doi: https://doi.org/10.1101/2024.01.23.576944; this version posted January 25, 2024. The copyright holder for this preprint (which was not certified by peer review) is the author/funder, who has granted bioRxiv a license to display the preprint in perpetuity. It is made available under aCC-BY 4.0 International license.

Figure S3. **V1 layer delineation for example penetration (B3) based on power spectrum (A), local coherence (B), instantiations power changes (C), CSD changes (D), unit density (E), spike spread (F), spike duration (G) and spike peak-trough ratio (H).**

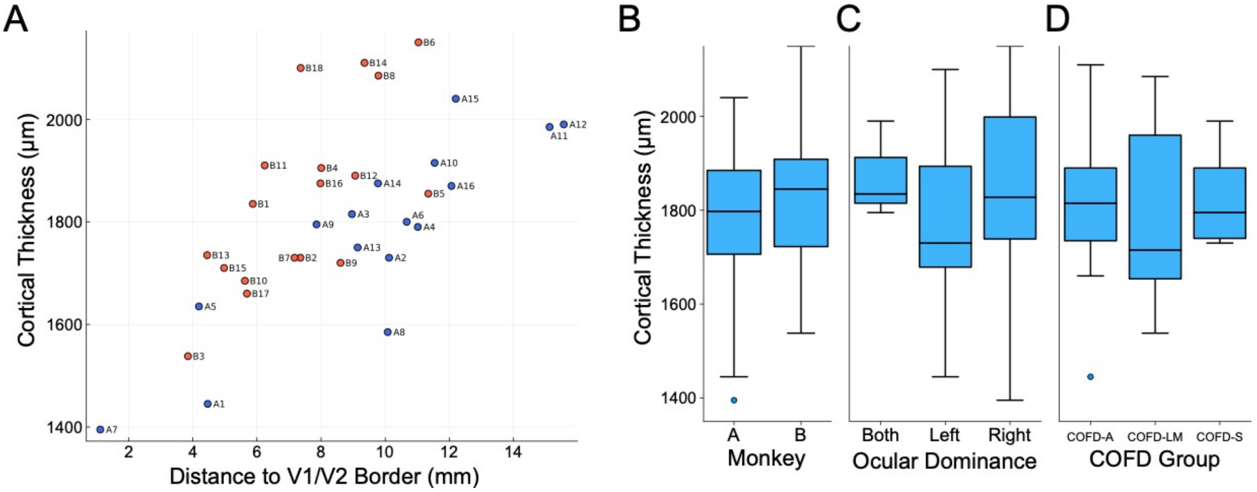

Figure S4. **Thickness of primary visual cortex**. (**A**) The distance was measured from the location of penetration perpendicular to V1/V2 border. Here, all penetrations except A17 (penetration location information lost) were included. (**B-D**) Distributions of cortical thickness across two monkeys, different ocular dominance columns, and COFD groups. No significant differences were found in each category (Kruskal-Wallis Test, $p>0.05$). COFD-A: Achromatic ON/OFF domains; COFD-LM: L- and M-cone ON/OFF domains; COFD-S: S-cone ON/OFF domains.

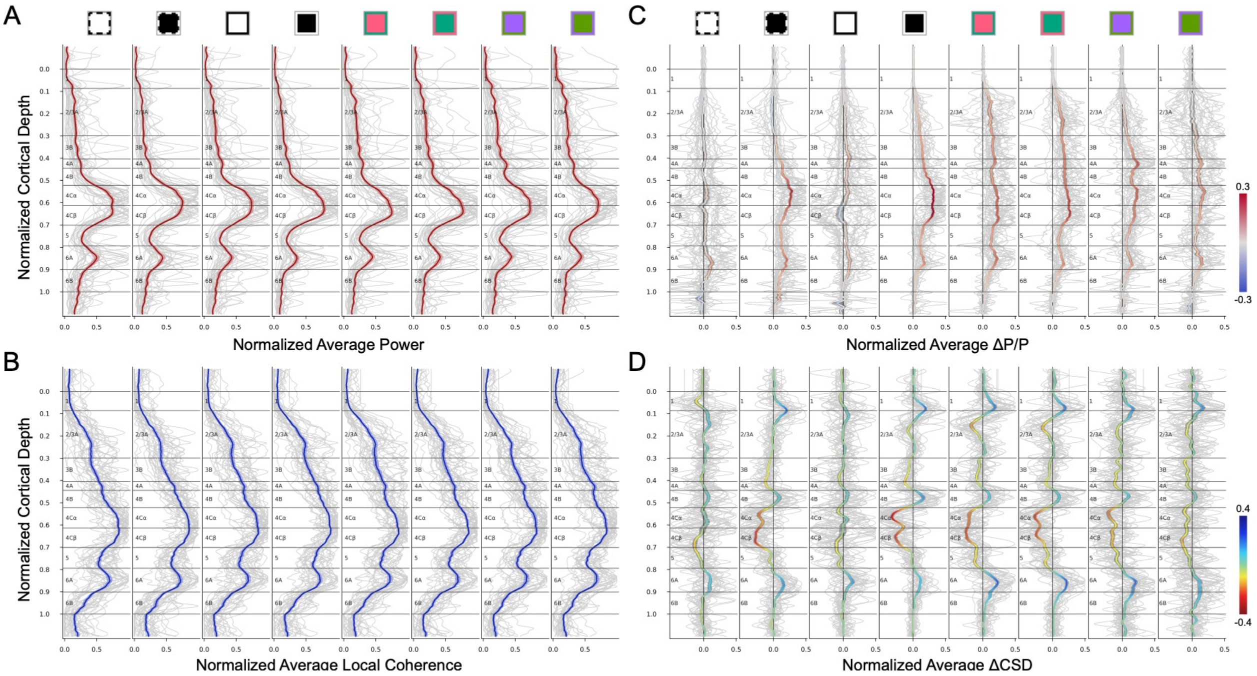

bioRxiv preprint doi: https://doi.org/10.1101/2024.01.23.576944; this version posted January 25, 2024. The copyright holder for this preprint (which was not certified by peer review) is the author/funder, who has granted bioRxiv a license to display the preprint in perpetuity. It is made available under aCC-BY 4.0 International license.

Figure S5. **Average Metrics in V1 template**. (**A**) Normalized average power for each stimulus. Gray lines are for each penetration, and red lines and shaded ribbons show Mean±SEM. (**B**) Normalized average local coherence for each stimulus is shown similarly to (A). (**C**) Normalized average ΔP/P in time window: [30, 100]ms after stimulus onset. (**D**) Normalized average ΔCSD in the same time window of C. The color-mapped lines and shaded gray ribbons in C and D show Mean±SEM. Square Markers are the same as in the figures of this article.

bioRxiv preprint doi: https://doi.org/10.1101/2024.01.23.576944; this version posted January 25, 2024. The copyright holder for this preprint (which was not certified by peer review) is the author/funder, who has granted bioRxiv a license to display the preprint in perpetuity. It is made available under aCC-BY 4.0 International license.

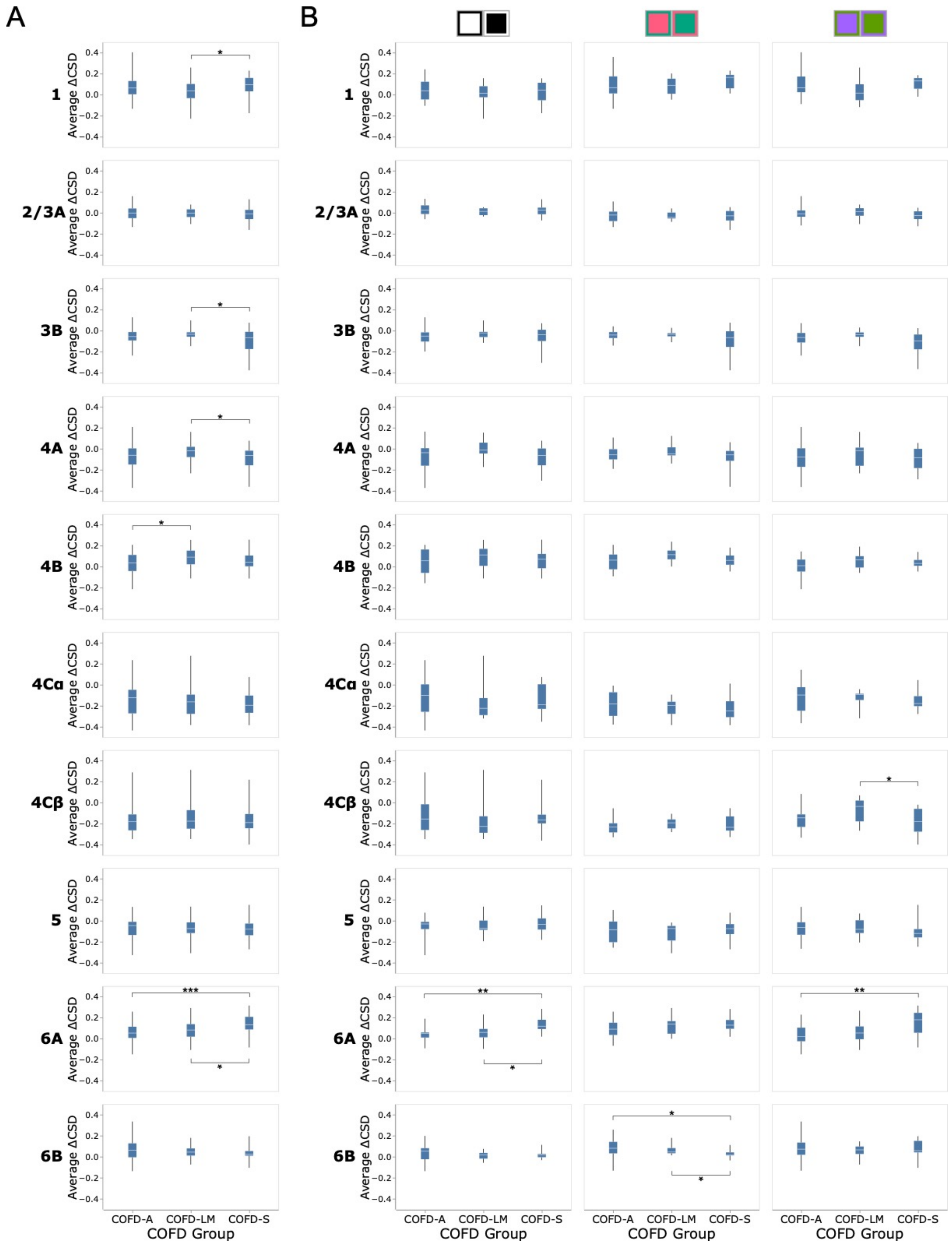

bioRxiv preprint doi: https://doi.org/10.1101/2024.01.23.576944; this version posted January 25, 2024. The copyright holder for this preprint (which was not certified by peer review) is the author/funder, who has granted bioRxiv a license to display the preprint in perpetuity. It is made available under aCC-BY 4.0 International license.

Figure S6. **Average normalized ΔCSD of each layer for different COFDs**. Averaging was applied for each penetration (gray lines in Figure S5D). COFD groups were compared in (**A**), and further compared under each stimuli group in (**B**). Statistical significance was represented as *: p<0.05, **: p<0.01, ***: p<0.001, Mann-Whitney U Test. COFD-A: Achromatic ON/OFF domains; COFD-LM: L- and M-cone ON/OFF domains; COFD-S: S-cone ON/OFF domains.

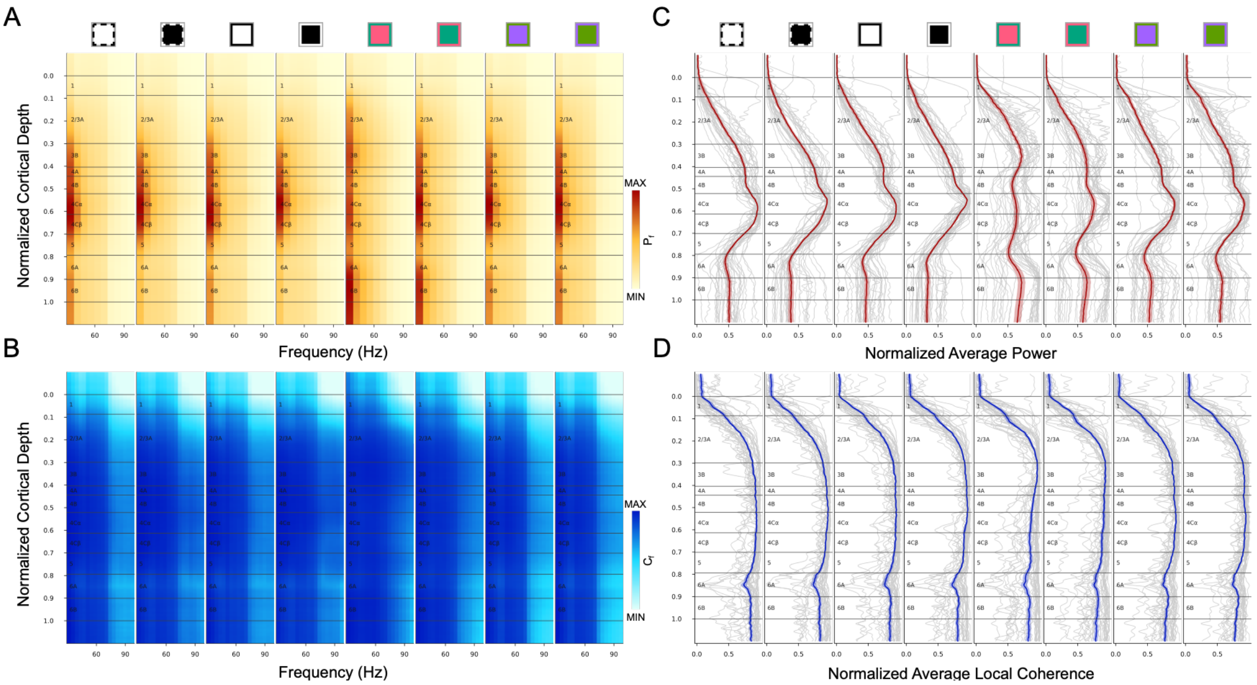

Figure S7. **Average metrics from gamma band LFP in V1 template**. (**A-B**) Average gamma band (30-100Hz) spectrum profile of power (A) and local coherence (B) across penetrations. The square markers and color bars were the same as the figures in this article. (**C-D**) Normalized average power and local coherence in the gamma band. Gray lines are for each penetration, color-lines and corresponding shaded ribbons show Mean±SEM.

bioRxiv preprint doi: https://doi.org/10.1101/2024.01.23.576944; this version posted January 25, 2024. The copyright holder for this preprint (which was not certified by peer review) is the author/funder, who has granted bioRxiv a license to display the preprint in perpetuity. It is made available under aCC-BY 4.0 International license.

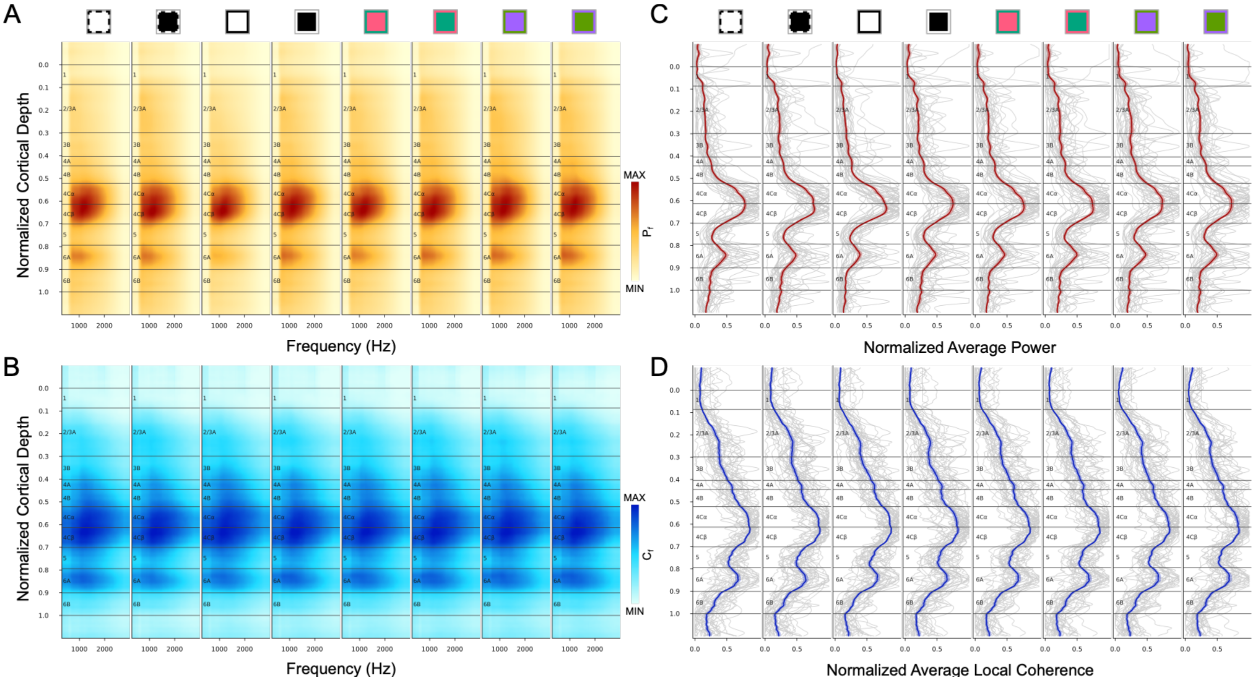

Figure S8. **Average metrics from baseline activity in V1 template**. (**A-B**) Average baseline AP spectrum profile of power (A) and local coherence (B) across penetrations. The square markers and color bars were the same as the figures in this article. (**C-D**) Normalized average baseline AP power and local coherence. Gray lines are for each penetration, color-lines and corresponding shaded ribbons show Mean±SEM.

bioRxiv preprint doi: https://doi.org/10.1101/2024.01.23.576944; this version posted January 25, 2024. The copyright holder for this preprint (which was not certified by peer review) is the author/funder, who has granted bioRxiv a license to display the preprint in perpetuity. It is made available under aCC-BY 4.0 International license.

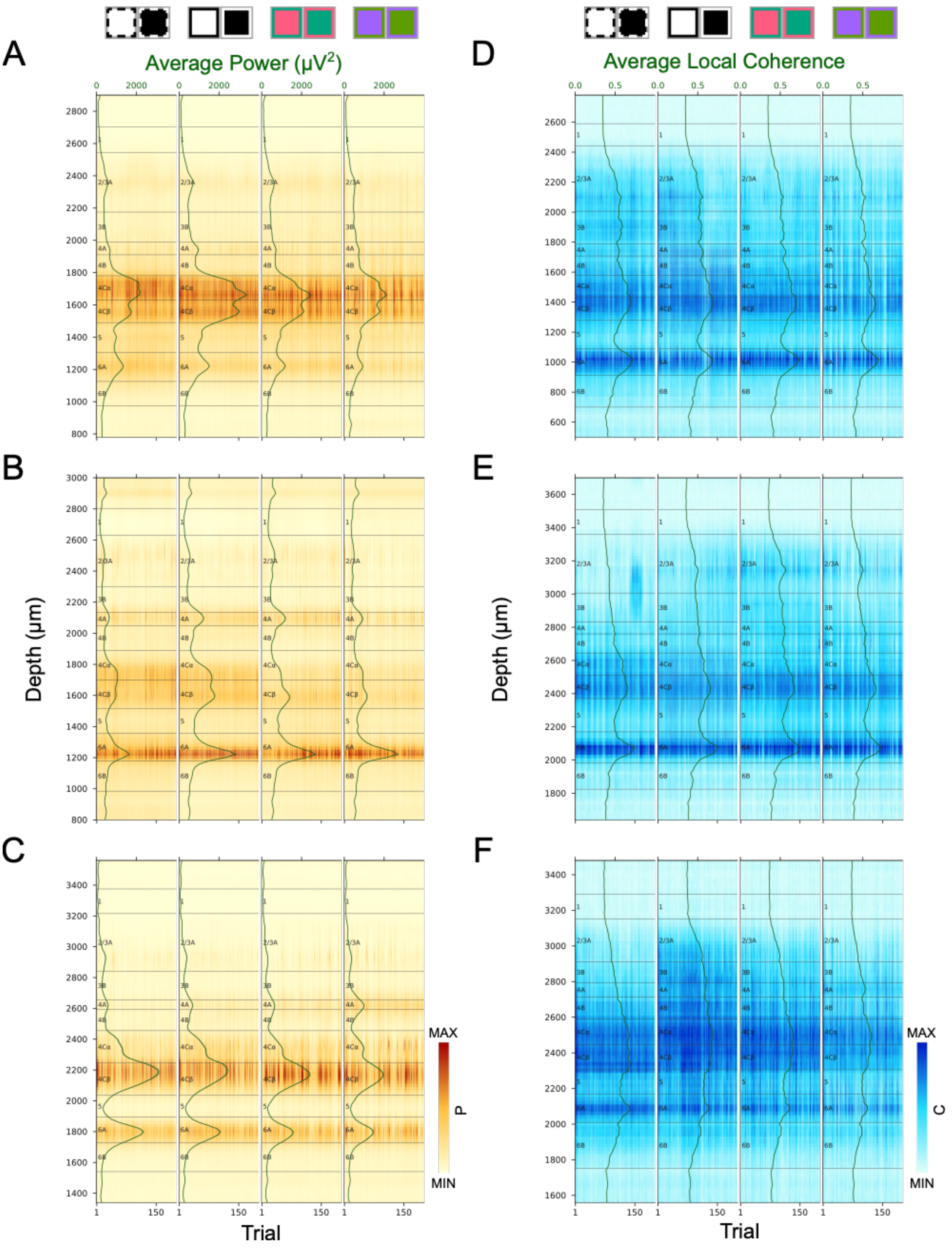

Figure S9. **AP power and local coherence averaged across frequencies for each trial**. (**A-C**) Powers for the same penetrations (A: A2, B: A3, C: B1) in Figure 5. (**D-F**) Local coherences for the same

bioRxiv preprint doi: https://doi.org/10.1101/2024.01.23.576944; this version posted January 25, 2024. The copyright holder for this preprint (which was not certified by peer review) is the author/funder, who has granted bioRxiv a license to display the preprint in perpetuity. It is made available under aCC-BY 4.0 International license.

penetrations (D: B12, E: B10, F: B3) in Figure 6. Power and local coherence profiles were independently color-mapped according to the minimum and maximum in each profile. The green lines and corresponding shaded ribbons showed the Mean±SEM across trials.

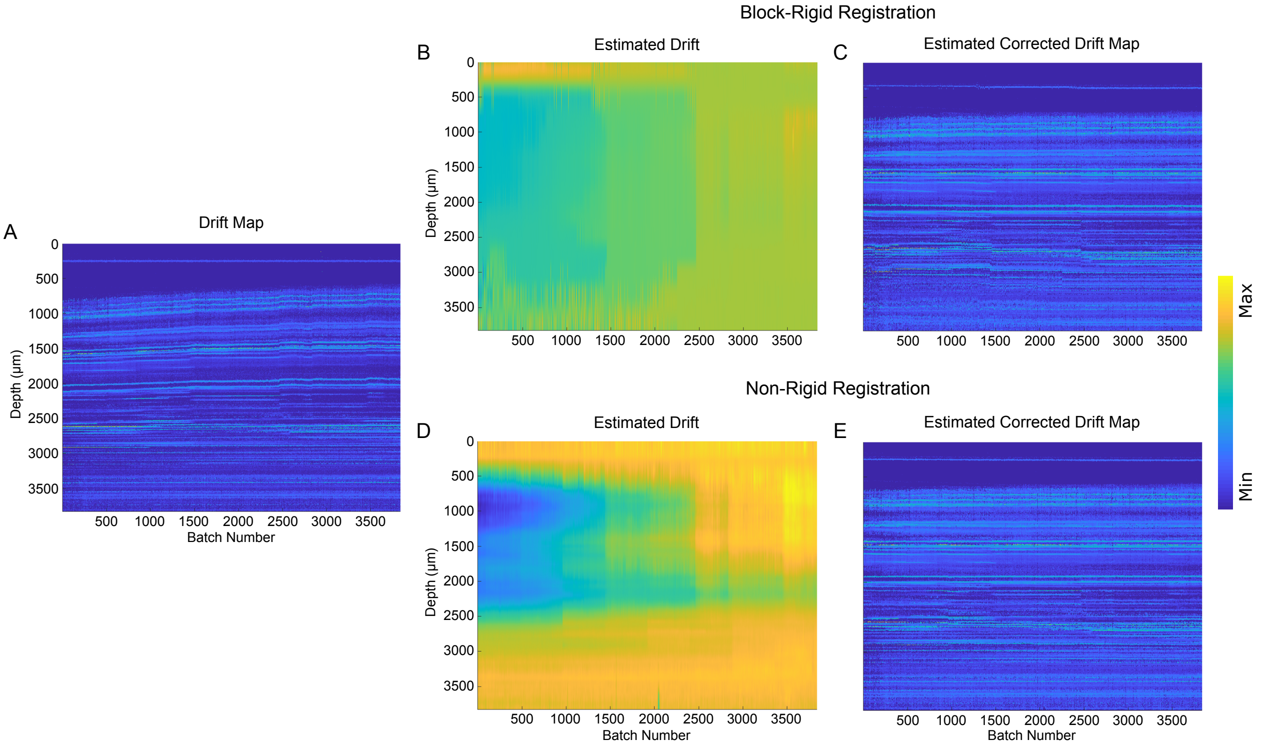

Figure S10. **Non-rigid drift correction**. (**A**) Probe drifting during the recording time for one penetration (B1) was visualized as average spike amplitudes shifting in consecutive batches (batch length: ~3.3 sec). (**B**) Drift estimated using the default method in Kilosort 3 (drift range: [-300, 300] μm, number of blocks: 13, drift range for each block: [-300, 300] μm). (**C**) Expected correction result after applying (`imwarp` function in MATLAB) the estimated drift in B on A. (**D**) Drift estimated using `imregdemons` function in MATLAB. (**E**) Expected correction result after applying (`imwarp` function in MATLAB) the estimated drift in D on A.
